# Forensic Features and Population Genetic Structure of Dong, Yi, Han, and Chuanqing Human Populations in Southwest China Inferred From Insertion/Deletion Markers

**DOI:** 10.3389/fgene.2020.00360

**Published:** 2020-04-30

**Authors:** Yubo Liu, Han Zhang, Guanglin He, Zheng Ren, Hongling Zhang, Qiyan Wang, Jingyan Ji, Meiqing Yang, Jianxin Guo, Xiaomin Yang, Jin Sun, Jinxing Ba, Dan Peng, Rong Hu, Lan-Hai Wei, Chuan-Chao Wang, Jiang Huang

**Affiliations:** ^1^Department of Forensic Medicine, Guizhou Medical University, Guiyang, China; ^2^Department of Anthropology and Ethnology, Institute of Anthropology, National Institute for Data Science in Health and Medicine, and School of Life Sciences, Xiamen University, Xiamen, China; ^3^Institute of Forensic Medicine, West China School of Basic Science and Forensic Medicine, Sichuan University, Chengdu, China; ^4^Faculty of Forensic Medicine, Zhongshan School of Medicine, Sun Yat-sen University, Guangzhou, China

**Keywords:** Investigator^®^ DIPplex Kit, Guizhou human population, Dong human population, Yi human population, Han human population, Chuanqing human population, forensic genetics, population structure

## Abstract

Guizhou province in southwest China has abundant genetic and cultural diversities, but the forensic features and genetic structure of Guizhou populations remain poorly understood due to the sparse sampling of present-day populations. Here, we present 30 insertion/deletion polymorphisms (InDels) data of 591 human individuals collected from four populations, Dong, Yi, Han, and Chuanqing residing in Guizhou. We calculated the forensic parameters of 30 InDel loci and found that this panel meets the efficiency of forensic personal identification based on the high combined power of discrimination, but it could only be used as a complementary tool in the parentage testing because of the lower combined probability of exclusion values. The studied populations are genetically closer related to geographically adjacent or linguistically related populations in southern China, such as the Tai-Kadai and Hmong-Mien speaking groups. The unrecognized ethnic Chuanqing people show an additional genetic affinity with Han Chinese, highlighting the role of possible military immigrations in their origin.

## Introduction

Insertion/deletion polymorphisms (InDels) are biallelic length polymorphisms based on insertion or deletion of one or more nucleotides in the genome. InDels are widely distributed across the human genome, with the total number estimated at approximately 2 million (Mills et al., 2011). InDels are considered to be ideal forensic markers with reduced amplicon size and low mutation rates (Sheng et al., 2018). In addition, InDels can be analyzed through simple PCR amplification and high-throughput electrophoresis, which are commonly used in forensic short tandem repeat (STR) analysis. Therefore, InDels have been increasingly explored and used in forensic genetics (Arenas et al., 2017; Chen et al., 2019; Pan et al., 2019).

Genetic structure analysis of East Asian populations plays an important role in understanding the migration patterns and genetic relationship of modern humans globally (Tian et al., 2018). Guizhou in southwest China is an ethnically diverse province with 18 native minorities including Miao, Bouyei, Dong, Sui, Gelao, Man, Mongolian, Hui, Yi, Qiang, and She. Therefore, Guizhou becomes an important region to explore the forensic features and comprehensive genetic structure of each nationality. However, limited genetic data of Guizhou groups have been published so far. We here used the first mature commercial kit (Investigator^®^ DIPplex Kit) (Turrina et al., 2011) to genotype the 30 InDels in 591 samples collected from the following four populations in Guizhou: an unrecognized ethnic group Chuanqing, Tai-Kadai speaking Dong, Sino–Tibetan speaking Yi, and Sinitic speaking Han Chinese. The aim of our study is first to explore the forensic efficiency of 30 InDel loci in personal identification and also infer the population genetic structure and history of the above four Guizhou populations.

Chuanqing people are an unrecognized ethnic group in Guizhou with a population at about 700,000. Most of the Chuanqing people live in Bijie Prefecture, Guizhou province, and speak a Sinitic language. There are two main hypotheses about the origin of Chuanqing in historical records: one suggests that they descended from Guizhou Turen (the indigenous people in Guizhou) and Han Chinese soldiers who were sent to Guizhou area in Ming dynasty, while the other suggests that Chuanqing are indigenous people in Guizhou without significant contribution from Han Chinese-related populations (Li, 2011). But there are no genetic researches on the Chuanqing people so far.

The Dong group, also known as Kam people, is the 10th largest ethnic minority group in China, accounting for a population of 2.9 million. Dong are native people of Guizhou and have the largest population at about 1.6 million in Tongren and Yuping Dong autonomous county in eastern Guizhou than in any other region of China. The language of Dong people belongs to the Tai-Kadai language family. The ancestor of southern Dong people may come from Guangxi Wuzhou and Guangdong Guangzhou. The population data of 30 autosomal InDels in Guangxi Dong group have been reported before (Wang et al., 2016), but there is no information about the genetic relationship between Guangxi Dong and Guizhou’s relevant populations.

The Yi ethnic group is the seventh largest ethnic minority group of China with about 8.7 million people according to the 2010 National Population Census^1^. The Yi people have lived in southwest China, such as Sichuan, Yunnan, and Guizhou provinces as early as before the pre-Qin dynasty. The Yi people have different branches and various cultural diversities in different areas, for example, they have six dialects. We here genotyped the 30 InDel loci in Guizhou Yi individuals, constructed the phylogenetic trees with Yi groups from different areas, analyzed and verified whether there are any genetic differences among Yi people in different regions.

The Han Chinese, with the world’s largest population at about 1.3 billion, make up over 92% of China’s vast population. The archeological and historical records suggest that Han Chinese can trace their ancestry to Huaxia tribes and possibly to the Neolithic Yan Huang tribes (Proto–Sino–Tibetan speakers) along the Upper and Middle Yellow River in northern China. After originating in the north (Zhao et al., 2015), the Han culture expanded southward by demic diffusion into the regions originally inhabited by the populations related to present-day speakers of Tai-Kadai, Austronesian, Austroasiatic, and Hmong-Mien languages (Fei, 1999; Ge et al., 1997). Previous analyses of genome-wide single-nucleotide polymorphism (SNP) array data have revealed that there is genetic substructure in Han Chinese populations with the main pattern corresponding to a general “north–south” cline (Chen et al., 2009; Xu et al., 2009). However, since Guizhou is a mountainous and multi-ethnic province, it is interesting to test what the genetic structure of Han Chinese in Guizhou looks like due to a long time of geographic isolation and genetic admixture with other surrounding indigenous populations. Previous studies of autosomal STR (Yang et al., 2017), X-STR (Chen et al., 2018), and Y-STR alleles (Sun et al., 2019) in Guizhou Han have been conducted, but the InDel data have not been reported before.

The Qiagen Investigator DIPplex Kit (Qiagen, Hilden, Germany) of 30 autosomal InDel loci is the first mature commercial InDels kit for forensic identification. For the past few years, vast amounts of genetic data have been published based on this kit from worldwide populations, such as African (Hefke et al., 2015), European (Friis et al., 2012; Kis et al., 2012), American (Saiz et al., 2014; Martinez-Cortes et al., 2016), Asian (Wang et al., 2014; Li et al., 2018; Zhang et al., 2019), and so on. However, very few reports on InDel markers are published from Guizhou groups. For the first time, we genotyped the 30 InDel loci in 591 unrelated individuals from four Guizhou groups Chuanqing, Dong, Yi, and Han using the Investigator DIPplex kit. We obtained the first batch of population data and calculated the forensic parameters. Next, we merged two reference datasets and applied various analysis methods to infer the genetic differentiation and genetic relationship between the studied populations and other previous published reference populations to shed more light on the genetic background of the Guizhou populations.

## Materials and Methods

### Sample Collection, DNA Extraction, and Quantification

**FIGURE 1:**
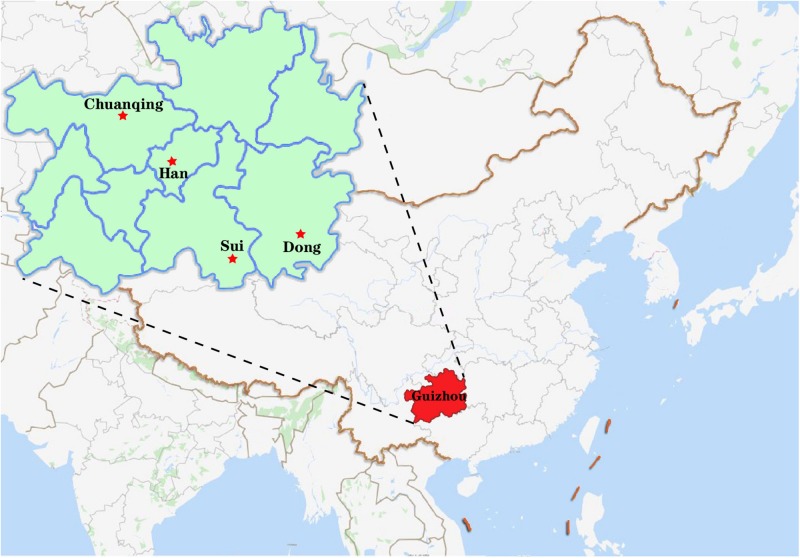
The geographical locations of four newly genotyped populations in this study.

A total of 591 unrelated individuals’ peripheral blood samples were gathered from four groups living in Guizhou province with informed consent, including 148 Dong, 152 Yi, 200 Han, and 91 Chuanqing individuals. A geographical map of the four ethnic groups is shown in Figure 1. The Ethics Committee of Guizhou Medical University approved all procedures involved in our study’s purpose, sample collections, experimental design, and so on. We have strict screening criteria about the studied unrelated individuals. In this study, those participants are indigenous people of Guizhou without blood relationships with each other. They have non-consanguineous marriages for over three generations. We collected the samples according to the following details: (1) both parents and grandparents being Dong, Yi, Han, and Chuanqing; (2) the mother tongues used are Dong, Yi, Han, and Chuanqing languages; (3) all participants from the same village or owning the same family names are needed to check with previously included subjects to avoid including close relatives; (4) in the past three generations, there are no documented ancestors from other ethnic groups. Human genomic DNA was extracted using the Chelex-100 (Walsh et al., 1991) and quantified (0.5–1.0 ng/ml) for amplification. For reference populations, we collected 15,180 individuals’ allele frequency data from 93 worldwide populations (Supplementary Figure S1), and the detailed sample size and geographic information were presented in Supplementary Table S1. Within the 93 populations, we found the raw genotype data of 6,561 individuals of 47 populations are publicly available from literature, and we downloaded the raw data for subsequent analysis. We analyzed the allele frequency and raw genotype data to infer if different types of data will give consistent results or not.

### DNA Amplification and Genotyping

We performed the amplification of 30 InDel loci with 12.5 μl volume reaction under the guidance of the manufacturer’s recommendations from Investigator DIPplex Kit (Qiagen, Hilden, Germany). The multiplex PCR was carried out on the GeneAmp^®^ PCR System 9700 Thermal Cycler (Applied Biosystems, Foster City, CA, United States). Amplification products were subsequently separated using capillary electrophoresis on an ABI 3500 Genetic Analyzer (Applied Biosystems, Foster City, CA, United States). The PCR products electropherograms were analyzed utilizing GeneMapper v3.2 software (Applied Biosystems) using DNA 9948 (Qiagen) and ddH_2_O as a positive control and negative control, respectively, accompanying every panel of DNA amplification and genotyping.

### Statistical Analysis

The Linkage Disequilibrium (LD) analysis was conducted based on raw genotype data by SNPAnalyzer Software v2.0 (Istech, South Korea) (Yoo et al., 2008). Subsequently, allele frequency and forensic statistical parameters such as match probability (PM), polymorphism information content (PIC), probability of exclusion (PE), discrimination power (PD), and typical paternity index (TPI) were calculated by STR Analysis for Forensics (STRAF) online software (Gouy and Zieger, 2017). The values of Hardy–Weinberg equilibrium (HWE), observed heterozygosity (Ho), expected heterozygosity (He), and p values of LD were estimated using the Arlequin software v3.5 (Excoffier and Lischer, 2010). Using PHYLIP Software v3.5^2^, we calculated Nei’s genetic distances based on allele frequencies of the 30 InDels between studied groups and 93 worldwide reference populations. We used R Statistical Software v3.0.2 to plot the heatmaps. The principal component analyses (PCA) of two different datasets were conducted by STRAF (Gouy and Zieger, 2017) and MVSP Software v3.22 (Kovach, 2007) based on raw genotype and allele frequency, respectively. We set the tolerance of eigenanalysis at 1E-007 when running the PCA. We used Genepop Software v4.0 (Rousset, 2008) to calculate the pairwise Fst distances based on raw genotype of 51 populations. Two phylogenetic neighbor-joining (NJ) trees were reconstructed by MEGA software v5.0 (Kumar et al., 2016) using genetic distance matrices based on two different population datasets, namely, Nei values of 97 groups and Fst values of 51 groups. To provide more clear genetic admixture pattern and infer the detailed ancestry component composition between studied populations and reference populations, we used the raw genotype dataset of 51 populations to run STRUCTURE analysis in STRUCTURE version 2.3.4.21 (Pritchard et al., 2000). We set the parameter to run 15 replicates from *K* = 2 to *K* = 8 with 10,000 burn-ins and 10,000 MCMC under the “LOCPRIOR” model in STRUCTURE analysis.

## Results

### Linkage Disequilibrium, Forensic Parameters, and Allele Frequency Distribution

We present the raw data of 30 InDel loci for our four newly genotyped populations of 591 individuals in Guizhou in Supplementary Table S2. We first carried out LD analysis. The degree of LD among the 30 InDel loci was illustrated in the form of the inverted triangle-like shape made of 435 small squares (Supplementary Figure S2). The various degrees of shades of red and white in the small square areas indicate the levels of the linkage between loci. Red color denotes a high level of linkage. Subsequently, we quantified the results of LD analysis by *r*^2^-values (data not listed) and *p*-values (Supplementary Table S3). There is no significant linkage between pairwise InDel loci for our four studied groups with *r*^2^ < 0.8 and *p* < 0.00167 after Bonferroni correction (0.05/30 = 0.00167). As shown in Supplementary Table S4, the loci HLD40, HLD70, and HLD81 in Dong, HLD67, and HLD81 in Yi, HLD4, and HLD77 in Chuanqing, and HLD6 in Han deviated from Hardy–Weinberg equilibrium (HWE). But the range of P values is acceptable after Bonferroni correction (*p* < 0.05/30). The results indicated that we can estimate both the forensic characteristics of the single locus and the combined forensic efficiency indices in our following analysis using our newly genotyped InDel data of Dong, Yi, Han, and Chuanqing.

The allele frequencies and forensic parameters are presented in Supplementary Table S4. The insertion allele frequencies for Dong, Yi, Han, and Chuanqing populations range from 0.0970 (HLD39) to 0.9340 (HLD118), 0.0493 (HLD111) to 0.9145 (HLD118), 0.0875 (HLD111) to 0.9300 (HLD118), and 0.0930 (HLD39) to 0.9120 (HLD99), respectively. The insertion allele frequency differences between loci are huge, which are even more than one order of magnitude. We observed the same results from other East Asian populations (Du et al., 2019; He et al., 2019a, b). On the contrary, the allele frequency distribution is relatively balanced in European and American populations (Kis et al., 2012; Saiz et al., 2014), indicating that the panel of 30 InDels is informative for population substructure dissection and ancestral information inference at the continental level. The polymorphism information content (PIC) values of Dong, Yi, Han, and Chuanqing range from 0.1024 to 0.3748, from 0.0893 to 0.3746, from 0.1217 to 0.3750, and from 0.1475 to 0.3750, respectively. The 22 PIC values out of the 30 loci are over 0.3 in Dong, Han, and Chuanqing groups, accounting for more than 73% of Dong, Han, and Chuanqing samples, while 21 PIC values are over 0.3 in the Yi group, accounting for 70% of Yi samples. The PIC values of some InDel markers are relatively small in the studied populations, such as HLD118, HLD111, HLD99, HLD39, and HLD64, suggesting that they might not be suitable for forensic studies in East Asian populations. The average values of PIC for Dong, Yi, Han, and Chuanqing are 0.3142, 0.3150, 0.3191, and 0.3182, respectively. Since InDels are di-alleles, the 30 InDels are less polymorphic than STR (Iyavoo et al., 2019; Lin et al., 2019; Wen et al., 2019). The Ho values for Dong, Yi, Han, and Chuanqing samples are in the ranges of 0.1149–0.5541, 0.0977–0.5526, 0.1400–0.5450, and 0.1539–0.5604, respectively. Additionally, the number of the power of discrimination (PD) values larger than 0.5 among 30 InDel loci is 22 in Dong, 23 in Yi, 24 in Han, and 22 in Chuanqing. However, all the values of the power of exclusion (PE) of 30 InDels are smaller than 0.3 for the four studied populations. The combined powers of exclusion (CPE) and combined powers of discrimination (CPD) for Dong, Yi, Han, and Chuanqing groups are 0.9741 and 1.0000, 0.9754 and 1.0000, 0.9755 and 1.0000, and 0.9767 and 1.0000, respectively. Based on the standard of 0.9999, this panel of 30 InDel loci meets the efficiency of forensic personal identification, but it could only be used as a complementary tool in the parentage testing because of lower CPE values.

**FIGURE 2:**
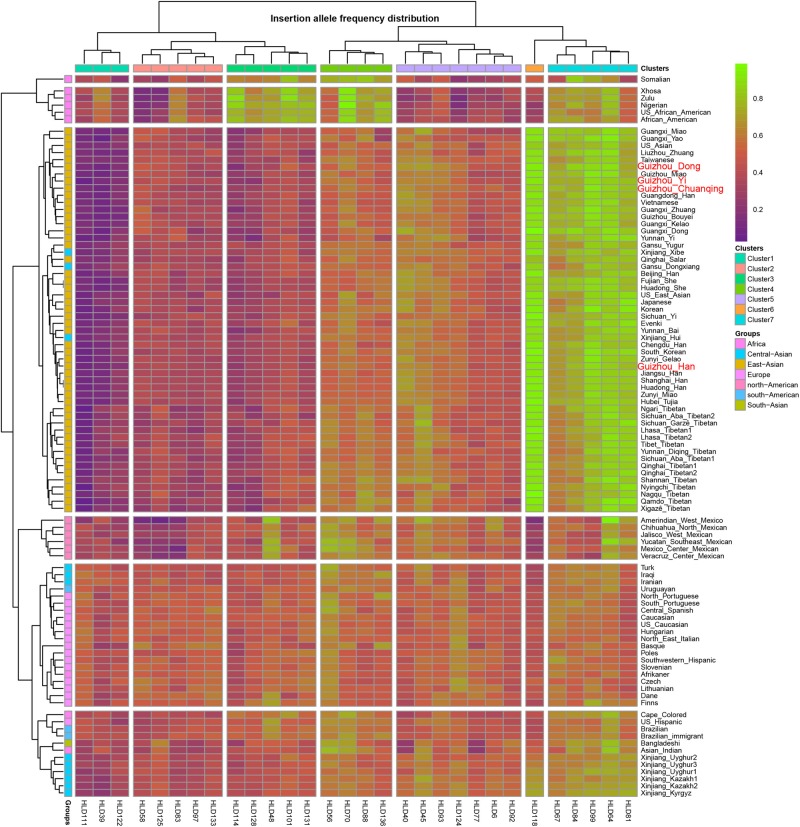
Heatmap on the basis of the insertion allele frequency distributions for Guizhou Dong, Yi, Han, and Chuanqing groups and 93 worldwide reference populations.

As we mentioned before, the allele frequency distributions of the 30 InDels are obviously different in different continental populations. We constructed a heatmap based on insertion allele frequencies of the 30 markers among the four studied populations and 93 worldwide populations, as shown in Figure 2. We conducted the clustering analysis of the 30 loci in 97 populations simultaneously. We found that the 30 InDel loci are divided into seven clusters (1∼7) as shown on the top line of the heatmap. Cluster 6 contains only one locus of HLD118. The 97 populations are divided into three large clusters: cluster I contains six African populations, cluster II mainly comprises of East Asian populations, cluster III mainly contains European, American, and Uighur populations. Since the Investigator DIPplex Kit was designed and used for the first time in the European populations for forensic practices, the allele frequencies of 30 InDel loci are approximately 0.5 among the cluster III populations, showing high heterozygosity and polymorphic. The cluster 6 and cluster 7 including HLD118, HLD67, HLD84, HLD99, HLD64, and HLD81 loci show high allele frequencies in East Asia populations. Instead, the markers of clusters 1∼3 are observed at low allele frequencies, and only 11 markers included in cluster 4 and cluster 5 show high heterozygosity and polymorphic in East Asians. Furthermore, African groups have different characteristics, with the markers in cluster 3, cluster 4, and cluster 7 showing high allele frequencies while the other markers showing low allele frequencies. The allele frequencies of 30 InDel loci show significant differences in clusters 1∼3, cluster 5, and cluster 6 among studied populations and other continental populations. However, the allele frequencies of all markers in cluster 4 and cluster 5 are similar among different populations. The allele frequency differences between Asian, European, and American populations are valuable in understanding the population genetic dynamics and useful in forensic identifications. Thus, we can select different loci for various forensic applications and design suitable forensic kit for the corresponding populations (Chen et al., 2019; Martinez et al., 2019) for human personal identification, biogeographical ancestry-inferring (LaRue et al., 2014; Lan et al., 2019), etc.

### Genetic Affinity of Four Guizhou Populations With Southern Minorities

We first conducted a series of PCAs to expound the genetic background and population relationship between Dong, Yi, Han, and Chuanqing in Guizhou province and populations from different continents and language families.

In the PCA plots based on the raw genotype data of 30 InDels of 6,561 individuals from 47 populations (Supplementary Figure S3A), we observed 16.64% of the genetic diversities are visualized by the first three PCs. In the PC1 and PC2, East Asian populations are in a large cluster showing close genetic relationships. The North American populations are separated by PC1 in the lower right, while the European, Turkic-speaking, and Sinitic-speaking populations gathered into a mixed cluster at the top right in Supplementary Figure S3A. Turkic-speaking populations involved in this study including Xinjiang-Uyghur, Xinjiang-Kazakh, and Gansu-Yugur have West Eurasian-related genetic admixture (Feng et al., 2017), which can explain the observed genetic similarities. As shown in Supplementary Figure S3B, we made a more detailed classification of 51 groups and found the Sinitic-speaking populations at the top right are Xinjiang-Hui and the studied Dong, Yi, Han, and Chuanqing overlapping with other East Asians. The similar results of genetic relationship and population clusters of 51 groups can be observed at the heatmap of deletion allele frequencies in Supplementary Figure S4.

**FIGURE 3:**
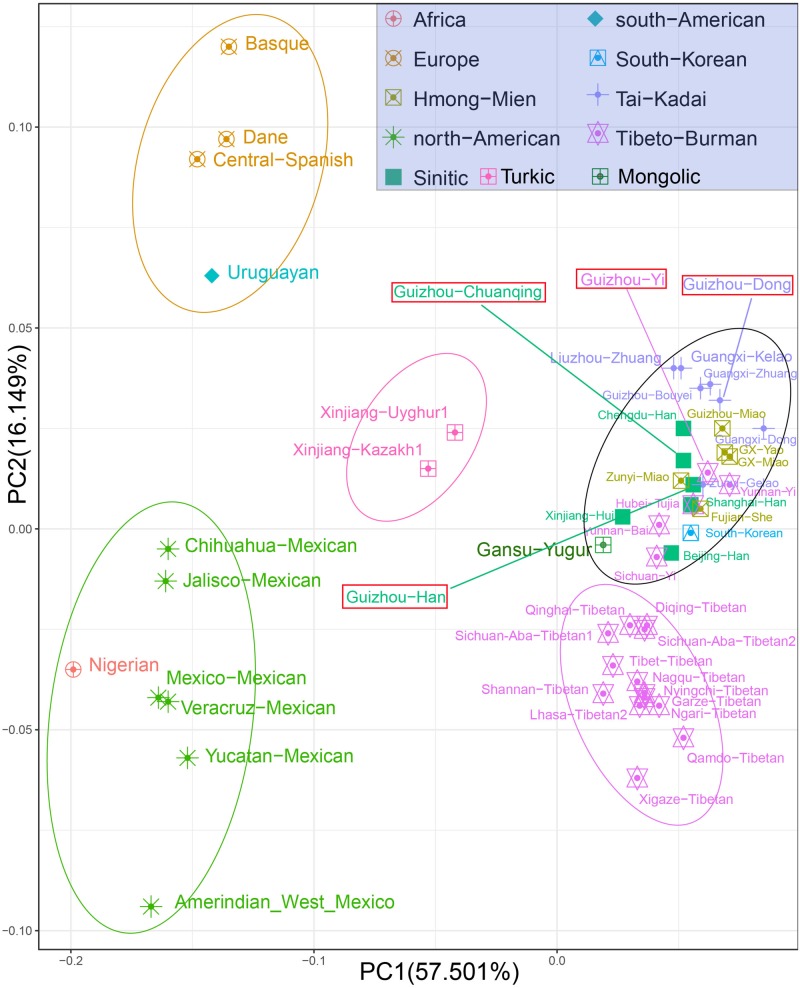
Principal component analysis among Guizhou Dong, Yi, Han, Chuanqing, and 47 reference populations on the basis of allele frequencies of 30 insertion/deletion polymorphisms (InDels).

At the population level, we carried out the PCA based on allele frequencies of 30 InDel loci of studied populations and other 47 reference populations. The first two PCs account for 73.65% of the variances among populations, as shown in Figure 3. We observed clear population clusters and genetic similarities, which further support the conclusion of the previous analysis. The 51 populations were labeled with 10 different shapes and colors: the African, European, North American, and South American were defined by geographic regions, and the East-Asian populations were further classified by different language groups such as Sinitic, Hmong-Mien, Tai-Kadai, Tibeto-Burman, Turkic, and South Koreanic. The North American populations and an African population clustered at the left lower corner; European groups and Uruguayan distributed in the left upper quadrant; and the studied four populations and 40 reference populations of China are in the right. We note that the North American–African cluster may not represent the genetic affinity of Americans and Africans but could be regarded as showing a relative position compared with East Asians. Besides, we only have one Nigerian population in the PCA, which could not reflect the abundant genetic diversity of Africans. The Uruguayan was plotted in the middle of European and Mexicans, which is consistent with their admixed population history. We observed three genetic subclusters within the East Asian populations: the two Turkic-speaking populations (Uyghur and Kazakh) clustered in the intermediate location between European and Asian populations; the 13 Tibetan minority groups clustered in the lower right corner; and the other Chinese mainland populations and Korean are in the right of center. We can explain the result of observed subclusters within East Asia as follows: Xinjiang Uyghur Autonomous Region is located in the heart of the ancient silk road and the Turkic-speaking groups in Xinjiang have a wide range of communicating and extensive admixture between Europe and Asia in history (Feng et al., 2017). Most of the Tibetan populations live at high altitudes with very limited contact with outside groups, making them genetically unique from other Chinese populations (Lu et al., 2016). The studied Dong, Yi, Han, Chuanqing groups clustered in the middle among the Chinese minorities, especially falling together with the Fujian She, Hubei Tujia, Shanghai Han, Zunyi Miao, Zunyi Gelao, Yunnan Yi, Guangxi Zhuang, and Guizhou Bouyei groups. The patterns of genetic similarity between studied populations and East Asian reference populations are roughly consistent with the geographical origin and linguistic affiliation. The PCA results of regional distribution pattern are in accordance with previous analysis based on the heatmap of allele frequency.

**FIGURE 4:**
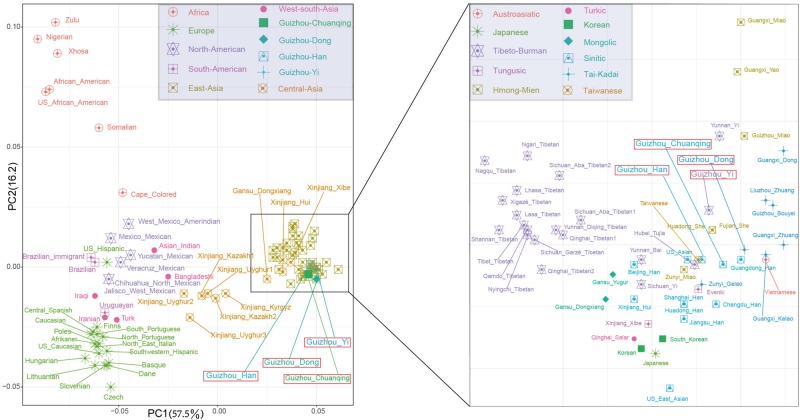
The two-dimensional plot of the first two principal components of principal component analyses among Guizhou Dong, Yi, Han, Chuanqing, and 93 worldwide reference populations based on allele frequencies of 30 insertion/deletion polymorphisms (InDels).

To further investigate the genetic relationships between studied groups and more reference populations from different language families and geographical regions, we co-analyze Dong, Yi, Han, and Chuanqing data with a new allele frequency dataset composing of 15,180 individuals from 93 worldwide populations. We initially carried out the PCA among 97 populations at the population level. The first two PCs show a total of 73.65% variance (Figure 4). We identified four main clusters from all of the populations on the PCA cline: the African populations in the upper left corner; the European populations in the lower left corner; the American populations in the middle; and the Asian populations in the most right. Our studied four populations fell into the inside of scatterplot of Asian populations grouped with Guangdong Han, Fujian She, Huadong She, Taiwanese, and Hubei Tujia.

**FIGURE 5:**
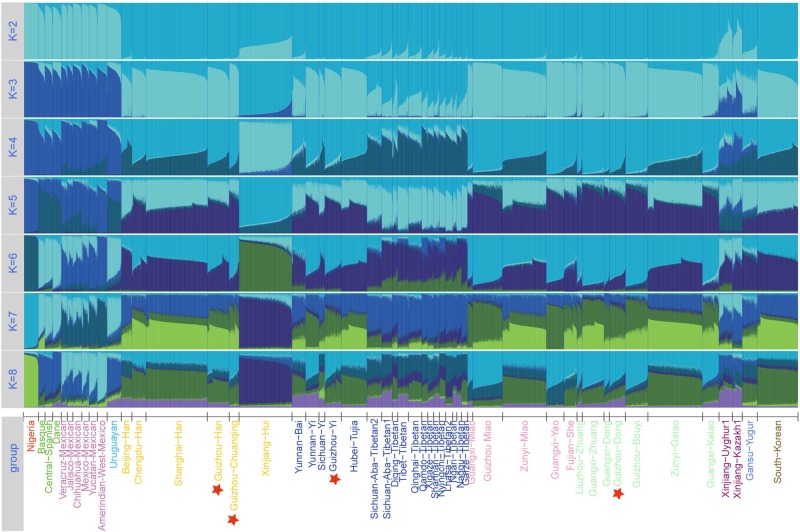
Structure analysis for the full-loci dataset with k ranging from 2 to 8 of the 51 populations.

Furthermore, to further investigate the internal structure of ancestral components of 51 groups at the individual level, we conducted STRUCTURE analysis based on the raw genotype of 7,152 individuals and set the range of *K*-values from 2 to 8. As shown in Figure 5, from the variations of color composition and verification results of each group from an online program STRUCTURE HARVESTER implementing the Evanno method^3^, the best *K*-value was observed at *K* = 4. When *K* > 4, we cannot find any further substructures in the 51 populations. At *K* = 2∼4, we observed that the groupings of 51 groups according to the proportions of various ancestry components are in accordance with geographic patterns. When *K* = 3, the Nigerians, European groups, American groups, and East Asian groups are distinguished because of the different ancestry compositions. At *K* = 4, the unique ancestry components of Uyghur, Kazakh, Yugur, Xinjiang Hui, and Tibetan groups are within the general East Asian pattern, and the other East Asian populations including our four studied groups (Dong, Yi, Han, and Chuanqing) show similar ancestral proportions with each other. Dong, Yi, Chuanqing, and Han of Guizhou have the largest difference in ancestral components with Nigerians, followed by European groups, American groups, and then the Xinjiang Hui, Turkic-speaking, and Tibetan groups. Hence, InDel marks can be used as a forensic practice tool to infer geographic ancestral components.

**FIGURE 6:**
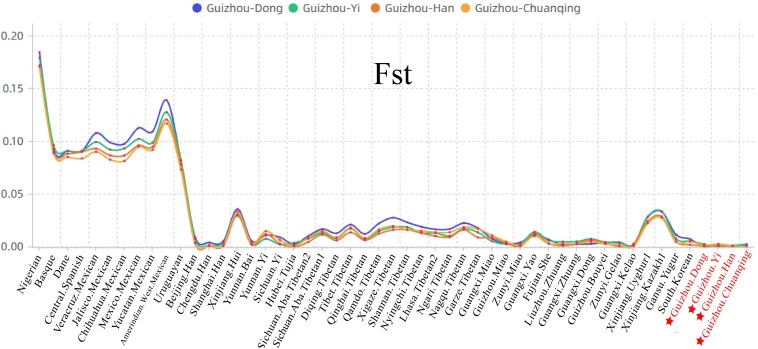
Line chart on the basis of the pairwise Fst genetic distances among Guizhou Dong, Yi, Han, Chuanqing, and 47 previously studied populations.

**FIGURE 7:**
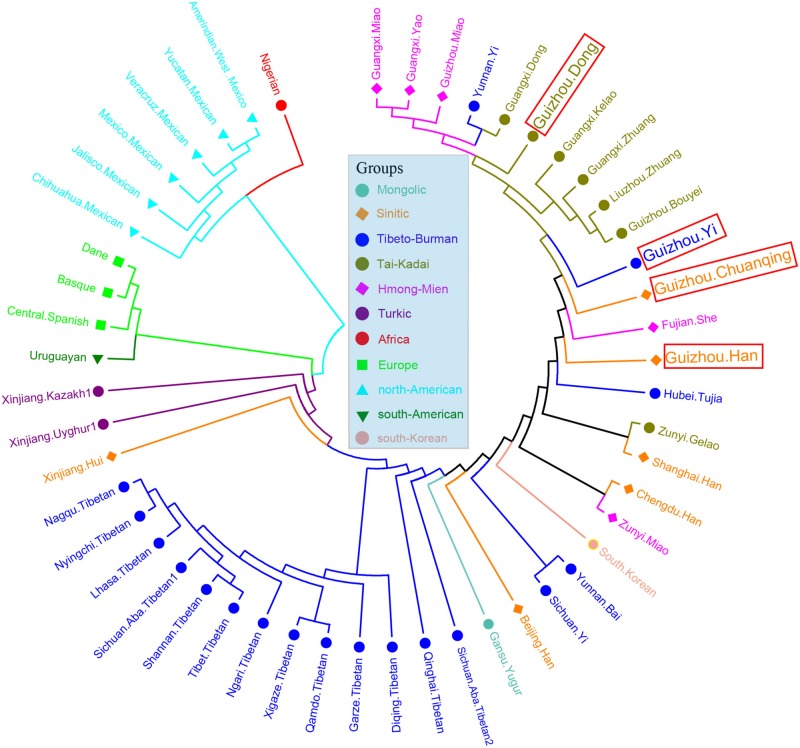
Phylogenetic relationship reconstruction by neighbor-joining algorithm among the 51 worldwide populations based on the pairwise Fst genetic distances.

We then calculated the pairwise Fst genetic distances between the studied populations and other 47 reference populations to confirm the observed genetic similarities. As shown in Figure 6 and Supplementary Table S5, the higher Fst values mean greater divergence between pairwise populations, and the line chart suggests that the maximum values are observed in using Nigerians for the comparison, followed by the European and American populations, then the Xinjiang and Tibetan populations. The result of Fst distances shows that the Dong, Yi, Han, and Chuanqing groups have close genetic affinities with linguistically or geographically close populations, especially the Chengdu Han, Zunyi Gelao, Hubei Tujia, Liuzhou Zhuang, and Guangxi Gelao. Finally, to more intuitively understand the genetic clustering among the 51 populations, we reconstructed the phylogenetic tree *via* an NJ algorithm based on the Fst genetic matrix. The identified three main branches of the NJ tree correspond well to the continental regions. As shown in Figure 7, the studied four groups clustered with East Asian populations except for Xinjiang and Tibetan populations. We found the strong correlations between genetics and linguistics within Hmong–Mien- and Tai–Kadai-speaking populations. The studied Guizhou Dong clustered with Tai–Kadai-speaking populations, especially with the populations from Guangxi, such as Guangxi Dong, Kelao, and Zhuang.

**FIGURE 8:**
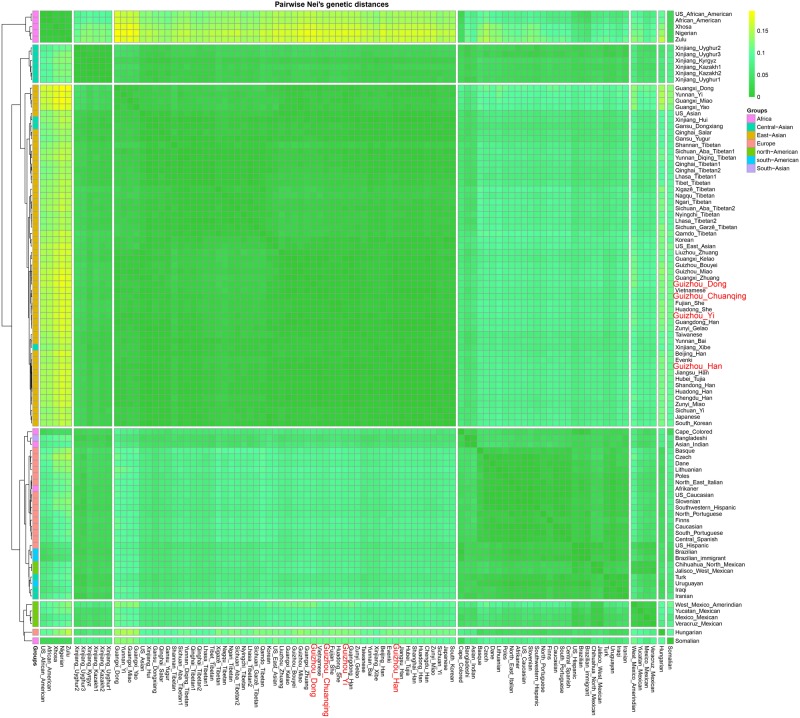
Heatmap on the basis of the pairwise Nei’s genetic distances of 30 deletion and insertion polymorphism (DIP) loci among 97 worldwide populations conducted with R Statistical Software.

We calculated the Nei’s genetic distances based on the allele frequency dataset composing of 15,180 individuals from 93 worldwide populations and constructed an NJ tree to offer more detailed information for genetic similarities and dissimilarities. The values of Nei’s distances between studied populations and other reference groups are shown in Supplementary Table S6. We here list values of the two closest populations with each of our four studied groups: Dong (Guangdong Han, 0.004238; Guangxi Kelao, 0.004285); Yi (Guizhou Miao, 0.003207; Zunyi Miao, 0.003313); Han (Zunyi Gelao, 0.000971; Hubei Tujia 0.001211); and Chuanqing (Guangxi Kelao, 0.002916; Hubei Tujia 0.003114). Furthermore, we identified that the values of Nei’s genetic distances are generally lower between the studied Dong, Yi, Chuanqing, with Guizhou Han (Dong 0.004313, Yi 0.002674, Chuanqing 0.003096) than with other ethnic minorities, showing a geographical affinity probably due to the frequent intermarriage between ethnic minorities and Han in Guizhou (Wu and Jiang, 2010; Lu and Zhang, 2014). Next, our pairwise Nei’s genetic distances were visualized in a heatmap plot. As shown in Figure 8, four-color areas are separated in the horizontal direction. The largest genetic distances are observed between our investigated four populations and Africans, followed by Americans, then the Europeans and Turkic-speaking Asians. Our studied populations are closest to bio-geographically adjoining populations.

**FIGURE 9:**
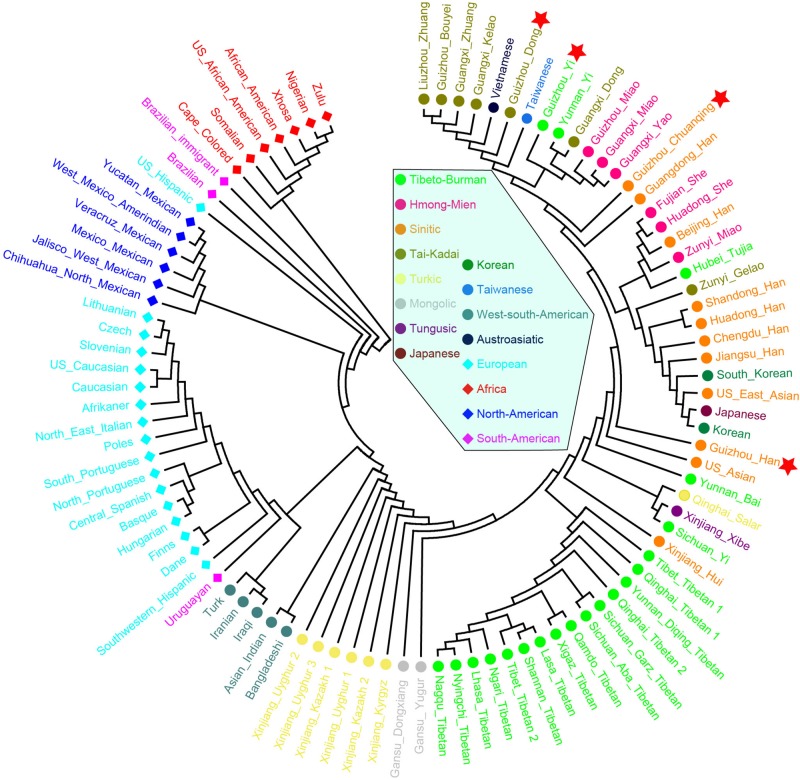
Phylogenetic relationship reconstruction by neighbor-joining algorithm among the 97 worldwide populations based on the pairwise Nei’s genetic distances.

Finally, to further understand the genetic relationships of four studied Guizhou populations with a larger dataset, the phylogenetic tree between studied populations and other 93 worldwide reference populations was reconstructed based on Nei’s genetic distance using the NJ method (Figure 9). Three main clusters and one admixed clade are identified clearly: African cluster, North American cluster, European cluster, and East Asian cluster. The subclusters of admixed clades consisted of Tibetan, Mongolian, and Turkic-speaking populations except for Qinghai Salar, showing a noteworthy genetic homogeneity compared with other geographical and linguistic populations. The four studied populations are scattered and intermingled within the East Asian populations. The Dong and Yi in Guizhou clustered together with Tai–Kadai- and Hmong–Mien-speaking populations in Guangxi and Guizhou, Yunnan Yi, and Taiwanese in southern China. The Chuanqing people are in an intermediate position between the above southern China cluster and Han Chinese, indicating the possible genetic influence from Han Chinese.

## Discussion

We genotyped the 30 InDel loci in 591 unrelated individuals from four Guizhou groups, Chuanqing, Dong, Yi, and Han using the Investigator DIPplex Kit. We reported the first batch of population data and calculated the forensic parameters. We found that this panel meets the efficiency of forensic personal identification based on the high combined power of discrimination (>0.9999), but it could only be used as a complementary tool in the parentage testing because of the lower combined probability of exclusion values. We also observed the allele frequency differences between Asian, European, and American populations, which remind that those markers can be used in various forensic applications and designing suitable forensic kit for the corresponding populations. We note that a newly developed panel of genotyping 50 InDels (AGCU InDel 50 kit) has been designed for Chinese populations. Liu et al. (2020) conducted the study in three Chinese ethnicities (Hainan Han, Hainan Li, and Zunyi Gelao groups) showing a higher capacity for the application of this InDel 50 kit in forensic medicine. Especially the CPD and CPE of AGCU InDel 50 kit were higher than those of the Investigator DIPplex kit. Meanwhile, the authors found that 22 loci showed significant different allele frequencies in the three studied populations. Obviously, the increasing number of InDel genetic markers has great potential for individual recognition and biogeographical ancestry inference.

Han Chinese are genetically substructured in a “north–south” cline inferred from autosomal genome-wide data with southern Han Chinese showing affinity with Tai–Kadai- and Hmong–Mien-speaking southern minorities in southern China (Chen et al., 2009; Hugo Pan-Asian Snp Consortium, 2009; Xu et al., 2009). However, from the paternal Y chromosomal perspective, southern Han Chinese except for Pinghua Han in Guangxi are genetically closer to northern Han Chinese compared with southern minorities (Wen et al., 2004). The Han Chinese in Guizhou are genetically closer to other Han Chinese groups all over China compared with southern minorities (Dong, Miao, Tujia, and Zhuang) based on the Y-STR data (Sun et al., 2019). Our analysis based on InDels is consistent with that inferred from autosomal genome-wide data that Guizhou Han show affinity with both other southern Han Chinese and also southern minorities, which combined with Y chromosomal evidence supporting that the demic diffusion of Han culture from north to south was dominated by males.

In the PCA and genetic distance-based NJ tree analysis above, our newly studied Guizhou Dong clustered with Tai–Kadai-speaking populations, especially the populations from Guangxi, such as Guangxi Dong, Kelao, and Zhuang. This result seems to support the hypothesis that the ancestor of southern Dong people may come from Guangxi Wuzhou. We detected that there are genetic substructures within Yi ethnic groups. The Yi ethnic groups from different areas are far away from each other as shown in the NJ tree, which can also be seen from the Fst genetic distances of Sichuan Yi and Yunnan Yi with Guizhou Yi (Sichuan Yi 0.001955 and Yunnan Yi 0.007291). The result is consistent with previous surveys from ethnography that the Yi people have different branches and various cultural diversities in different areas.

There are many hypotheses about the origin of Chuanqing people in Guizhou province. For example, one suggests that they descended from Guizhou Turen and Han Chinese soldiers who were sent to Guizhou area in Ming dynasty, while one suggests that Chuanqing are indigenous people in Guizhou. Based on the analysis of 30 InDels, we found that Chuanqing people have a closer genetic relationship with geographically adjacent Guangxi Kelao and Guizhou Han, which tends to support the role of possible military immigrations in their origin. However, we note that we could not be able to draw a clear conclusion about the origin of Chuanqing people based on 30 InDels. Further research based on genome-wide array genotyping or whole genome sequencing may shed more light on the origin of Chuanqing.

## Conclusion

In this study, we first investigated the forensic efficiency of the 30-InDel panel in Dong, Yi, Han, and Chuanqing residing in Guizhou, southeast China. We provided the first batch of forensic reference genotype data and forensic parameters of 30 autosomal InDel loci in 591 individuals from the above four populations. The results of the forensic statistical analysis demonstrate that this Investigator DIPplex Kit is sufficiently powerful for forensic individual identification and limited exclusive power for parental testing. We observed the genetic structure revealed by 30 InDels is generally consistent with geographical and linguistic classifications. In the future study, the whole-genome deep sequencing of represented populations with a large sample size should be carried out to provide a better understanding of the genetic structure and population history for Guizhou populations in southwest China.

## Data Availability Statement

The datasets generated for this study can be found in the Figshare https://figshare.com/articles/30_InDel_loci_genotyped_from_Dong_Yi_Han_and_Chuanqing_in_Southwest_China/9937811.

## Ethics Statement

Our study was carried out according to the recommendations of the Guizhou Medical University Ethics Committee. The protocol was reviewed and approved by the Human and Ethics Committee of the Guizhou Medical University, China. All study participants provided written informed consent in accordance with the Declaration of Helsinki.

## Author Contributions

JH and C-CW designed this study. HaZ and C-CW wrote the manuscript. YL and HaZ conducted the experiment. GH, ZR, HoZ, QW, JJ, MY, JG, XY, JS, JB, DP, RH, L-HW, C-CW, and JH analyzed the results. All authors reviewed the manuscript.

## Conflict of Interest

The authors declare that the research was conducted in the absence of any commercial or financial relationships that could be construed as a potential conflict of interest.

## Footnotes

1 http://www.stats.gov.cn/tjsj/pcsj/rkpc/6rp/indexch.htm

2 http://evolution.genetics.washington.edu/phylip.html

3 http://taylor0.biology.ucla.edu/structureHarvester/

## Supplementary Material

FIGURE S1 Population size and geographical locations of the four studied populations and other worldwide reference populations. Colors indicate the population size.

FIGURE S2 The linkage disequilibrium schema among the 30 InDel loci of **(A)** Dong, **(B)** Yi, **(C)** Han, and **(D)** Chuanqing.

FIGURE S3 Principal component analyses (PCA) among 7152 individuals. **(A)** PCA based on the first three components. **(B)** PCA based on the first two components and the more detailed classification.

FIGURE S4 Heatmap on the basis of the deletion allele frequency distributions for Guizhou Dong, Yi, Han, Chuanqing groups, and 47 worldwide reference populations.

TABLE S1 Population size, geographical locations and references information of the two datasets used in this study.

TABLE S2 Raw data for the 30 Indels in Dong, Yi, Han, and Chuanqing groups of Guizhou.

TABLE S3 The *p*-values of Linkage Disequilibrium for Guizhou Dong, Yi, Han, Chuanqing among 30 Indels detected in the Investigator DIPplex amplification system.

TABLE S4 Allele frequencies and forensic parameters for the 30 Indels in Dong, Yi, Han, and Chuanqing groups of Guizhou.

TABLE S5 The pairwise Fst genetic distances between the studied and 47 worldwide populations on the basis of the raw data of 30 InDels.

TABLE S6 The Nei’s genetic distances between the Guizhou Dong, Yi, Han, and Chuanqing and 93 worldwide reference populations.

## References

[B1] Arenas M. Pereira F. Oliveira M. Pinto N. Lopes A. M. Gomes V. (2017). Forensic genetics and genomics: Much more than just a human affair. *PLoS Genet.* 13:e1006960. 10.1371/journal.pgen.1006960 28934201 PMC5608170

[B2] Chen J. Zheng H. Bei J. X. Sun L. Jia W. H. Li T. (2009). Genetic structure of the Han Chinese population revealed by genome-wide SNP variation. *Am. J. Hum. Genet.* 85 775–785. 10.1016/j.ajhg.2009.10.016 19944401 PMC2790583

[B3] Chen L. Du W. Wu W. Yu A. Pan X. Feng P. (2019). Developmental validation of a novel six-dye typing system with 47 A-InDels and 2 Y-InDels. *Forensic Sci. Int. Genet.* 40 64–73. 10.1016/j.fsigen.2019.02.009 30776773

[B4] Chen P. He G. Zou X. Wang M. Jia F. Bai H. (2018). Forensic characterization and genetic polymorphisms of 19 X-chromosomal STRs in 1344 Han Chinese individuals and comprehensive population relationship analyses among 20 Chinese groups. *PLoS One* 13:e0204286. 10.1371/journal.pone.0204286 30235314 PMC6147642

[B5] Du W. Feng C. Yao T. Xiao C. Huang H. Wu W. (2019). Genetic variation and forensic efficiency of 30 indels for three ethnic groups in Guangxi: relationships with other populations. *PeerJ* 7:e6861. 10.7717/peerj.6861 31110924 PMC6501771

[B6] Excoffier L. Lischer H. E. (2010). Arlequin suite ver 3.5: a new series of programs to perform population genetics analyses under Linux and Windows. *Mol. Ecol. Resour.* 10 564–567. 10.1111/j.1755-0998.2010.02847.x 21565059

[B7] Fei X. T. (1999). *The Pattern of Diversity in Unity of the Chinese Nation.* Beijing: Central Univ. for Nationalities Press.

[B8] Feng Q. Lu Y. Ni X. Yuan K. Yang Y. Yang X. (2017). Genetic History of Xinjiang’s Uyghurs suggests bronze age multiple-way contacts in Eurasia. *Mol. Biol. Evol.* 34 2572–2582. 10.1093/molbev/msx177 28595347

[B9] Friis S. L. Borsting C. Rockenbauer E. Poulsen L. Fredslund S. F. Tomas C. (2012). Typing of 30 insertion/deletions in Danes using the first commercial indel kit–Mentype(R) DIPplex. *Forensic Sci. Int. Genet.* 6 e72–e74. 10.1016/j.fsigen.2011.08.002 21903497

[B10] Ge J. X. Wu S. D. Chao S. J. (1997). *Zhongguo yimin shi. The Migration History of China.* Fuzhou: Fujian People’s Publishing House.

[B11] Gouy A. Zieger M. (2017). STRAF-A convenient online tool for STR data evaluation in forensic genetics. *Forensic Sci. Int. Genet.* 30 148–151. 10.1016/j.fsigen.2017.07.007 28743032

[B12] He G. Adnan A. Rakha A. Yeh H. Y. Wang M. Zou X. (2019a). A comprehensive exploration of the genetic legacy and forensic features of Afghanistan and Pakistan Mongolian-descent Hazara. *Forensic Sci. Int. Genet.* 46 514–518. 10.1016/j.fsigen.2019.06.018 31257046

[B13] He G. Ren Z. Guo J. Zhang F. Zou X. Zhang H. (2019b). Population genetics, diversity and forensic characteristics of Tai-Kadai-speaking Bouyei revealed by insertion/deletions markers. *Mol. Genet. Genomics* 294 1343–1357. 10.1007/s00438-019-01584-6 31197471

[B14] Hefke G. Davison S. D’Amato M. E. (2015). Forensic performance of Investigator DIPplex indels genotyping kit in native, immigrant, and admixed populations in South Africa. *Electrophoresis* 36 3018–3025. 10.1002/elps.201500243 26404054

[B15] Hugo Pan-Asian Snp Consortium. (2009). Mapping human genetic diversity in Asia. *Science* 326 1541–1545. 10.1126/science.1177074 20007900

[B16] Iyavoo S. Afolabi O. Boggi B. Bernotaite A. Haizel T. (2019). Population genetics data for 22 autosomal STR loci in European, South Asian and African populations using SureID((R)) 23comp human DNA identification Kit. *Forensic Sci. Int.* 301 174–181. 10.1016/j.forsciint.2019.05.033 31167154

[B17] Kis Z. Zalan A. Volgyi A. Kozma Z. Domjan L. Pamjav H. (2012). Genome deletion and insertion polymorphisms (DIPs) in the Hungarian population. *Forensic Sci. Int. Genet.* 6 e125–e126. 10.1016/j.fsigen.2011.09.004 22014386

[B18] Kovach W. L. (2007). *MVSP-A MultiVariate Statistical Package for Windows, ver. 3.1.* Wales: Kovach Computing Services Pentraeth.

[B19] Kumar S. Stecher G. Tamura K. (2016). MEGA7: molecular evolutionary genetics analysis version 7.0 for bigger datasets. *Mol. Biol. Evol.* 33 1870–1874. 10.1093/molbev/msw054 27004904 PMC8210823

[B20] Lan Q. Shen C. Jin X. Guo Y. Xie T. Chen C. (2019). Distinguishing three distinct biogeographic regions with an in-house developed 39-AIM-InDel panel and further admixture proportion estimation for Uyghurs. *Electrophoresis* 40 1525–1534. 10.1002/elps.201800448 30758063

[B21] LaRue B. L. Lagace R. Chang C. W. Holt A. Hennessy L. Ge J. (2014). Characterization of 114 insertion/deletion (INDEL) polymorphisms, and selection for a global INDEL panel for human identification. *Leg. Med.* 16 26–32. 10.1016/j.legalmed.2013.10.006 24296037

[B22] Li L. P. (2011). On the Records of“ChuanQingRen”and the national origins and ethnic appellations of ancestors in guizhou chorography. *Guizhou Ethnic Stud.* 32 159–166.

[B23] Li Z. Xu J. Chen P. Yin C. Hu L. Huang H. (2018). Forensic efficiency and genetic divergence of 30 autosomal InDels in Chinese Han population from Jiangsu province. *Forensic Sci. Int. Genet.* 37 e17–e19. 10.1016/j.fsigen.2018.08.006 30153989

[B24] Lin S. W. Lam T. T. Ip S. C. Y. (2019). Population data of 23 autosomal STR loci in Hong Kong Chinese. *Forensic Sci. Int. Genet.* 39 e24–e25. 10.1016/j.fsigen.2018.11.018 30503808

[B25] Liu J. Du W. Wang M. Liu C. Wang S. He G. (2020). Forensic features, genetic diversity and structure analysis of three Chinese populations using 47 autosomal InDels. *Forensic Sci. Int. Genet.* 45:102227. 10.1016/j.fsigen.2019.102227 31865224

[B26] Lu D. Lou H. Yuan K. Wang X. Wang Y. Zhang C. (2016). Ancestral origins and genetic history of tibetan highlanders. *Am. J. Hum. Genet.* 99 580–594. 10.1016/j.ajhg.2016.07.002 27569548 PMC5011065

[B27] Lu G. Zhang Y. Q. (2014). A historical study of the interracial marriages in China. *J. Yunnan Norm. Univ.* 46 15–22.

[B28] Martinez J. Polverari F. S. Silva F. A. J. Braganholi D. F. Ferraz J. Gusmao L. (2019). Genetic characterization of 32 X-InDels in a population sample from Sao Paulo State (Brazil). *Int. J. Legal Med.* 133 1385–1388. 10.1007/s00414-018-01988-w 30612323

[B29] Martinez-Cortes G. Garcia-Aceves M. Favela-Mendoza A. F. Munoz-Valle J. F. Velarde-Felix J. S. Rangel-Villalobos H. (2016). Forensic parameters of the Investigator DIPplex kit (Qiagen) in six Mexican populations. *Int. J. Legal Med.* 130 683–685. 10.1007/s00414-015-1242-y 26233613

[B30] Mills R. E. Pittard W. S. Mullaney J. M. Farooq U. Creasy T. H. Mahurkar A. A. (2011). Natural genetic variation caused by small insertions and deletions in the human genome. *Genome Res.* 21 830–839. 10.1101/gr.115907.110 21460062 PMC3106316

[B31] Pan X. Liu C. Du W. Chen L. Han X. Yang X. (2019). Genetic analysis and forensic evaluation of 47 autosomal InDel markers in four different Chinese populations. *Int. J. Legal Med.* 10.1007/s00414-019-02059-4 [Epub ahead of print]. 30997570

[B32] Pritchard J. K. Stephens M. Donnelly P. (2000). Inference of population structure using multilocus genotype data. *Genetics* 155 945–959. 10835412 10.1093/genetics/155.2.945 PMC1461096

[B33] Rousset F. (2008). genepop’007: a complete re-implementation of the genepop software for Windows and Linux. *Mol. Ecol. Resour.* 8 103–106. 10.1111/j.1471-8286.2007.01931.x 21585727

[B34] Saiz M. Andre F. Pisano N. Sandberg N. Bertoni B. Pagano S. (2014). Allelic frequencies and statistical data from 30 INDEL loci in Uruguayan population. *Forensic Sci. Int. Genet.* 9 e27–e29. 10.1016/j.fsigen.2013.07.013 23953140

[B35] Sheng X. Bao Y. Zhang J. S. Li M. Li Y. N. Xu Q. N. (2018). Research progress on InDel genetic marker in forensic science. *Fa Yi Xue Za Zhi* 34 420–427. 10.12116/j.issn.1004-5619.2018.04.016 30465411

[B36] Sun H. Su K. Fan C. Long F. Liu Y. Sun J. (2019). Y-STRs’ genetic profiling of 1953 individuals from two Chinese Han populations (Guizhou and Shanxi). *Forensic Sci. Int. Genet.* 38 e8–e10. 10.1016/j.fsigen.2018.10.011 30392972

[B37] Tian J. Y. Li Y. C. Kong Q. P. Zhang Y. P. (2018). [The origin and evolution history of East Asian populations from genetic perspectives]. *Yi Chuan* 40 814–824. 10.16288/j.yczz.18-202 30369466

[B38] Turrina S. Filippini G. De Leo D. (2011). Forensic evaluation of the Investigator DIPplex typing system. *Forensic Sci. Int. Genet.* 3 e331–e332. 10.1016/j.fsigss.2011.09.028

[B39] Walsh P. Metzger D. Higuchi R. (1991). Chelex 100 as a medium for simple extraction of DNA for PCR-based typing from forensic material. *Biotechniques* 10 506–513. 1867860

[B40] Wang L. Lv M. Zaumsegel D. Zhang L. Liu F. Xiang J. (2016). A comparative study of insertion/deletion polymorphisms applied among Southwest, South and Northwest Chinese populations using Investigator((R)) DIPplex. *Forensic Sci. Int. Genet.* 21 10–14. 10.1016/j.fsigen.2015.08.005 26656953

[B41] Wang Z. Zhang S. Zhao S. Hu Z. Sun K. Li C. (2014). Population genetics of 30 insertion-deletion polymorphisms in two Chinese populations using Qiagen Investigator(R) DIPplex kit. *Forensic Sci. Int. Genet.* 11 e12–e14. 10.1016/j.fsigen.2014.03.017 24780854

[B42] Wen B. Li H. Lu D. Song X. Zhang F. He Y. (2004). Genetic evidence supports demic diffusion of Han culture. *Nature* 431 302–305. 15372031 10.1038/nature02878

[B43] Wen D. Yang Z. Sun S. Aliye K. Judes Z. M. M. Lan L. (2019). Genetic polymorphisms of new 22 autosomal STR loci in the Mongolian ethnic group. *Int. J. Legal Med.* 133 1405–1407. 10.1007/s00414-019-02111-3 31236678

[B44] Wu X. P. Jiang G. D. (2010). Analysis the relationship between Tunpu people and the local minoritis in contemporary from the perspective of Tunpu people’s intermarrying with local minorities. *Guizhou Ethnic Stud.* 31 58–65.

[B45] Xu S. Yin X. Li S. Jin W. Lou H. Yang L. (2009). Genomic dissection of population substructure of Han Chinese and its implication in association studies. *Am. J. Hum. Genet.* 85 762–774. 10.1016/j.ajhg.2009.10.015 19944404 PMC2790582

[B46] Yang M. Ren Z. Ji J. Zhou H. Zhang H. Dai J. (2017). Population genetic data and mutations of 22 autosomal STR loci in Guizhou Han population. *Forensic Sci. Int. Genet.* 29 e29–e30. 10.1016/j.fsigen.2017.03.008 28410948

[B47] Yoo J. Lee Y. Kim Y. Rha S. Y. Kim Y. (2008). SNPAnalyzer 2.0: a web-based integrated workbench for linkage disequilibrium analysis and association analysis. *BMC Bioinformatics* 9:290. 10.1186/1471-2105-9-290 18570686 PMC2453143

[B48] Zhang H. He G. Guo J. Ren Z. Zhang H. Wang Q. (2019). Genetic diversity, structure and forensic characteristics of Hmong-Mien-speaking Miao revealed by autosomal insertion/deletion markers. *Mol. Genet. Genomics* 294 1487–1498. 10.1007/s00438-019-01591-7 31312894

[B49] Zhao Y. B. Zhang Y. Zhang Q. C. Li H. J. Cui Y. Q. Xu Z. (2015). Ancient DNA reveals that the genetic structure of the northern Han Chinese was shaped prior to 3,000 years ago. *PLoS One* 10:e0125676 10.1371/journal.pone.0125676 PMC4418768 25938511

