# Supplementary figures and images for: Forensic Features and Population Genetic Structure of Dong, Yi, Han, and Chuanqing Human Populations in Southwest China Inferred From Insertion/Deletion Markers
Source: Front Genet. 2020 Apr 30;11:360. doi: 10.3389/fgene.2020.00360 (PMC7205039; doi:10.3389/fgene.2020.00360)

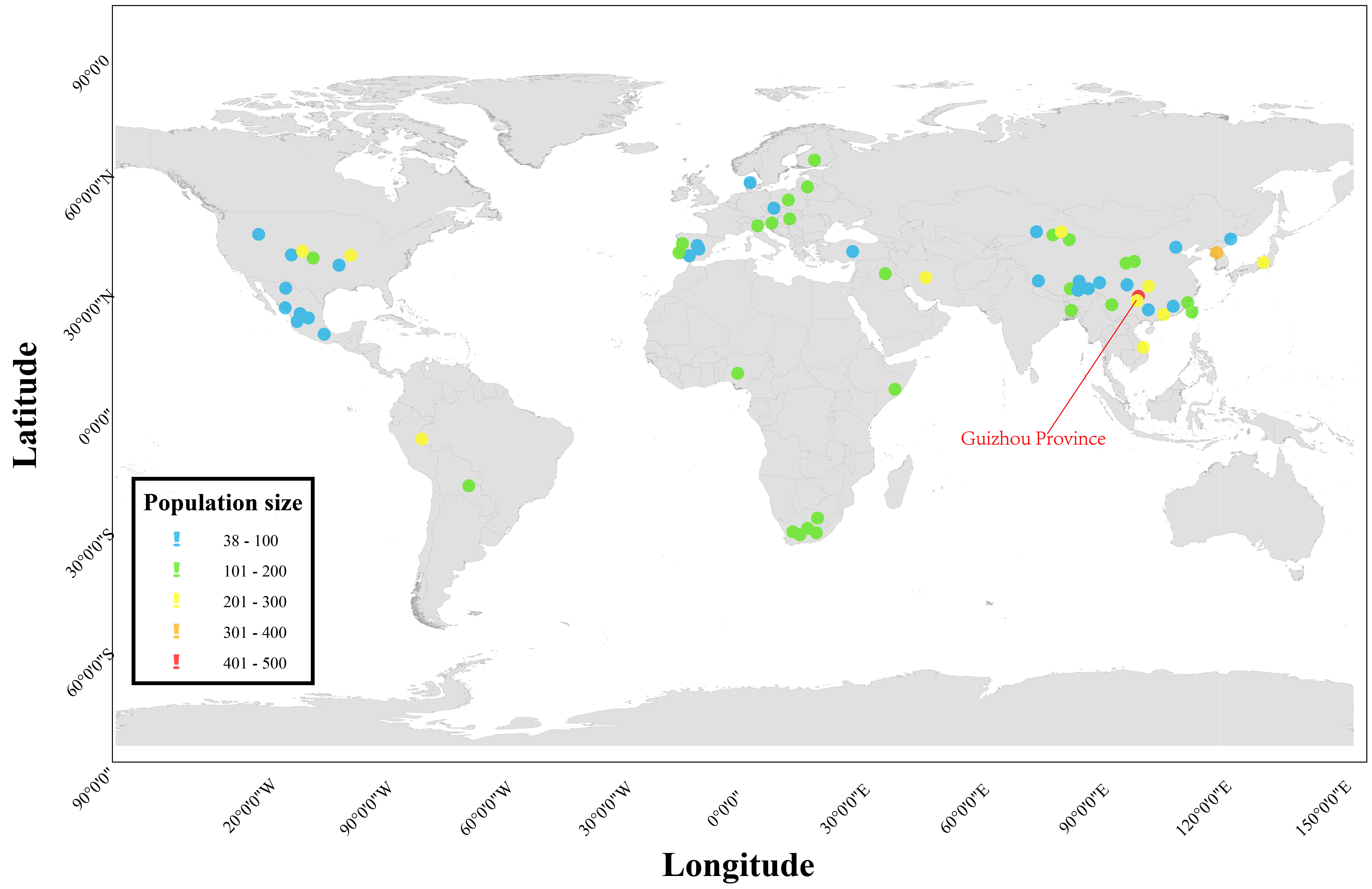

Supplement: FIGURE S1 — Population size and geographical locations of the four studied populations and other worldwide reference populations. Colors indicate the population size. [file Image_1.JPEG]

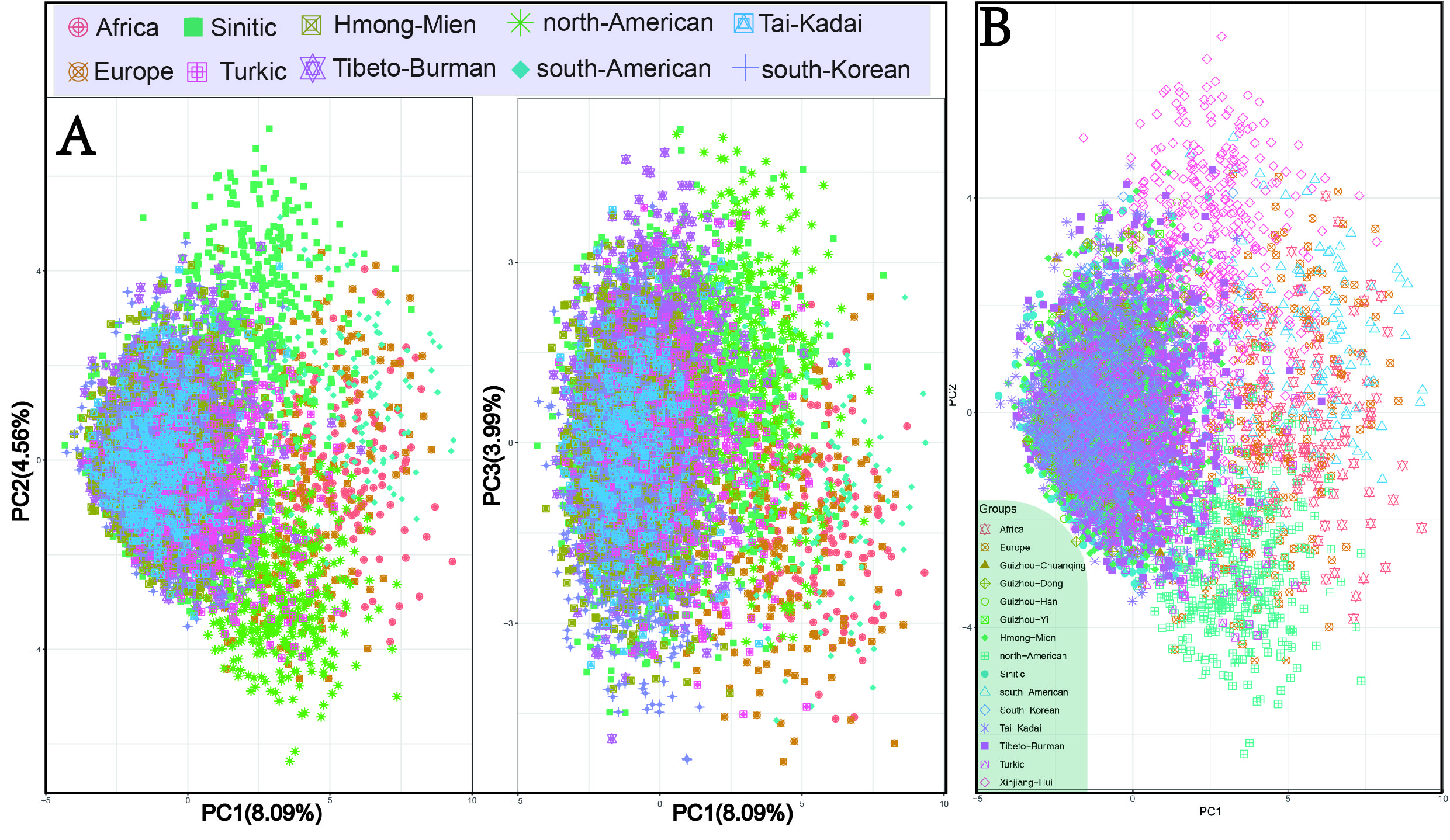

Supplement: FIGURE S3 — Principal component analyses (PCA) among 7152 individuals. (A) PCA based on the first three components. (B) PCA based on the first two components and the more detailed classification. [file Image_3.jpg]

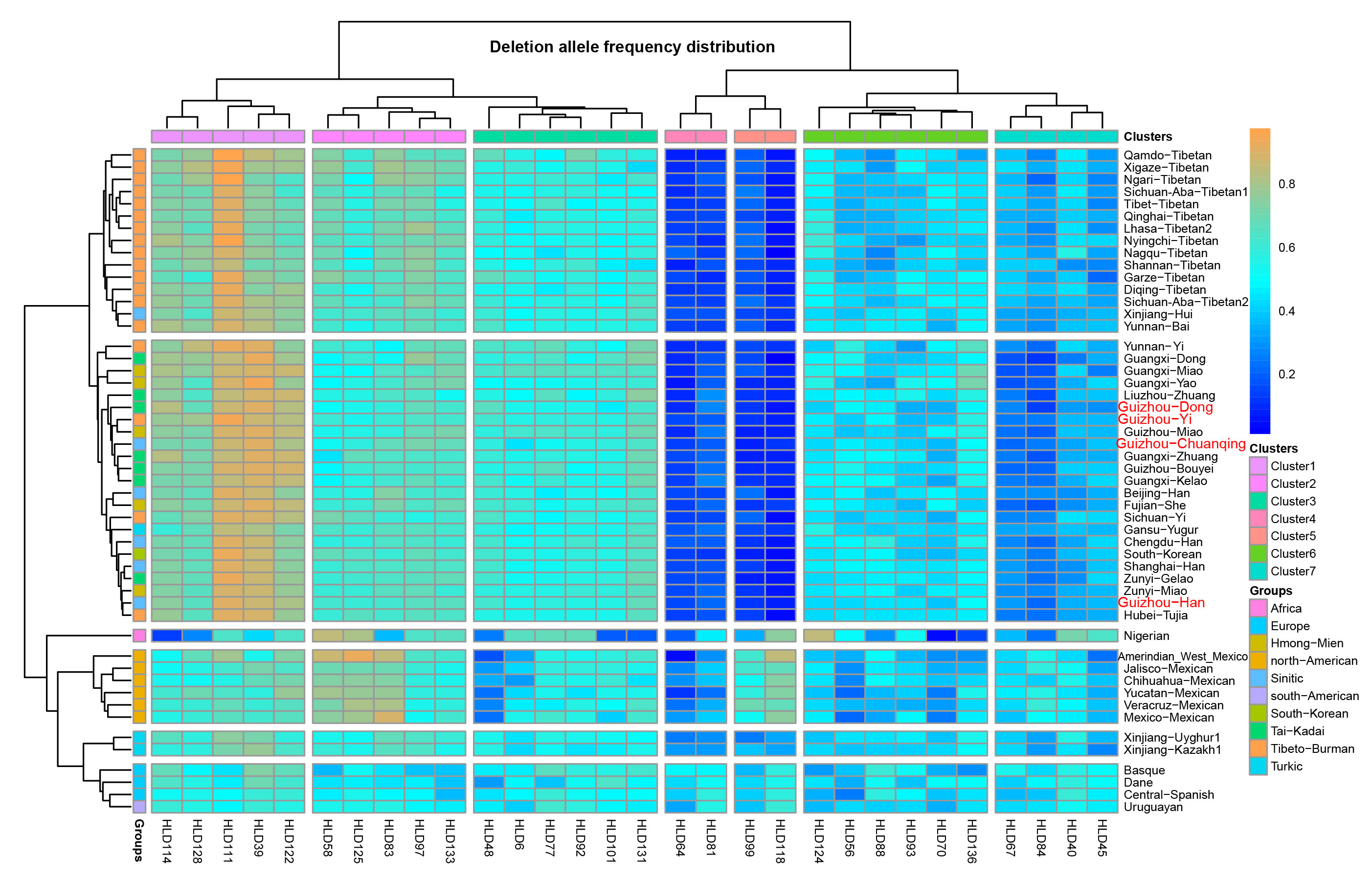

Supplement: FIGURE S4 — Heatmap on the basis of the deletion allele frequency distributions for Guizhou Dong, Yi, Han, Chuanqing groups, and 47 worldwide reference populations. [file Image_4.JPEG]
